# Psychographic predictors of intention to use cervical cancer screening services among women attending maternal and child health services in Southern Ethiopia: the theory of planned behavior (TPB) perspective

**DOI:** 10.1186/s12889-019-6745-x

**Published:** 2019-04-25

**Authors:** Fira Abamecha, Atsede Tena, Getachew Kiros

**Affiliations:** 1 Sidama Zone Health Department, SNNPR, Hawassa, Ethiopia; 2 Department of Health, Behavior and Society, Institute of Health, Faculty of Public Health, Jimma University, Ethiopia, PO Box 378, Jimma, Ethiopia

**Keywords:** Cervical cancer, Screening, Intention, TPB, Ethiopia

## Abstract

**Background:**

Detection of the premalignant forms cervical cancer through screening in the target age group is one of the effective strategies in the prevention of the disease. Nevertheless, the cervical cancer screening service use remains considerably low in Ethiopia. Indeed; promoting screening behaviors requires understanding the factors influencing women’s motivation towards the service. Our study has explored the psycho-graphic factors associated to intention to use cervical cancer screening among women visiting maternal and child health services in Southern Ethiopia, 2017.

**Methods:**

Institution based cross-sectional study was used employing 422 women’s age between 30 and 49 years old. A structured questionnaire was used to collect data on interviewer-administered basis. All assumptions of theory of planned behavior (TPB) were considered to measure intention, attitude, perceived social pressure and perceived ability to control circumstances against cervical cancer screening. Data were analyzed using statistical package for social sciences version 21.0. Multiple linear regression models were conducted to identify factors associated to intention to use cervical cancer screening. *P*-value less than 5% was considered to indicate significant association.

**Result:**

Four hundred and two (95%) of the respondents completed the interview. Knowledge of the disease signs, symptoms, risk factors and prevention methods was 162(41.4%). Knowledge about the disease and past screening experience were positively associated with intention to use cervical cancer screening (β = 0.145, 95% CI = [0.047, 0.170]) and (β = 0.098, 95% CI = [0.093, 1.001]) respectively. Further; standardized regression coefficient showed that all dimensions of TPB were positively associated to intention to use the services with perceived behavioral control (β = 0.297, 95% CI = [0.172, 0.343]), perceived social pressure (β = 0.248, 95% CI = [0.131, 0.301]) and attitude towards screening (β = 0.110, CI = [0.018, 0.158]).

**Conclusion:**

Overall; the intention to use cervical cancer screening was a function of attitude, perceived social pressure and perceived behavioral control confirming the hypothesis of the study. None of the socio-demographic variables were associated to intention. Health behavior change interventions should focus on increasing knowledge and empowering women that enable them to evaluate their control beliefs and develop ability against social norms and circumstances that compete with the use of cervical cancer screening services.

## Background

Cervical cancer is a global public health problem accounting the fourth most common cancer-affecting women worldwide. The world has estimated population of 2,716 million women aged 15 years and older who are at risk of developing cervical cancer. About 527,624 women are already diagnosed with cervical cancer and 265,672 die from the disease annually. About; 87% all deaths from cervical cancer occurs in sub-Sahara African countries [1]. The high prevalence of cervical cancer is also evident in the case of Ethiopia. There were 27.19 million women aged 15 years and older who are at risk of developing cervical cancer [2]. Every year; 7,095 Ethiopian women are diagnosed with cervical cancer and 4,732 die from the disease. It has been shown that the highest cancer-related mortality rate; comprising 16.5%; occurred among women in Ethiopian in 2015 [3].

Despite its proven importance, rates of attendance for screening programs vary widely, and are considerably low. The low prevalence of early cervical cancer screening and limited access to its treatments largely attributed to differences in diagnosis and subsequent mortality from the disease among high and low income countries. For instance; the screening uptake for cervical cancer in the three developing regions was only 6%, 12%, and 8.3% in South Africa, Bhutan, and Nigeria respectively [4–6]. It has been recorded that only 1% of eligible women received the cervical cancer screening services in Ethiopia with about 90% of them never had a pelvic examination at all [7]. The death from cervical cancer is found to be high among women who didn’t undergo screening and those who have sexual health risks like early sexual practices, multiple sexual partners and multiple pregnancies [8]. Furthermore; delayed treatment seeking behavior with advanced stage of the disease is common in developing countries which could markedly leads to diminished chance of success of treatment even with multiple modalities including brachytherapy, radiotherapy and chemotherapy [9].Understanding the competing and motivating factors affecting the CCS behavior among women in the context of Ethiopia helps to enhance the screening and treatment efforts. In addition to their limited quantity; most previous studies conducted in the Ethiopia narrowly emphasized on cognitive dimensions and accessibility of service related factors [10–12]. However, none of them has comprehensively addressed the complex normative dimensions and circumstances that importantly influence the women’s decision-making process and intention to use CCS. Hence, the study is the first of its kind in Ethiopia in applying the most widely applied and successfully studied behavioral theory to predict the intended use of cervical cancer screening services.

The Theory of Planned Behavior (TPB), which is an extension of the Theory of Reasoned Action (TRA), was developed by Ajzen in 1990. It is one of the most widely employed social-cognitive theories to understand the relationship between intentions and behavior [13]. According to the TPB, attitude towards a behavior, subjective norms and perceived behavioral control (PBC) determine intention which in turn predicts the behavior [14]. The TPB has been found to be the most parsimonious model in predicting intentions and various behavioral outcomes [15]. A meta-analysis showed that the TPB accounted for 39% of the variance in intentions and 27% of the variance in behavior across a broad spectrum of behaviors [16]. Further; TPB has also been applied to screening behaviors like breast and colorectal cancers screening behaviors [17]. It has been found to be effective in predicting cervical cancer screening (CCS) intention in different previous studies [18–20].

The current study has applied TPB due to the following important reasons. Women's decision-making is the result of intrapersonal factors (the process of decision-making reflects people's values and attitudes). To act in a specific direction requires that people behave in line with their motivation and attitudes. In addition, participating in screening services is a result of external factors that control or enhance the behavior. Personal factors like one’s confidence to cope with and overcome barriers related to availability, affordability and accessibility of screening services and social supports are all supposed to determine the extent to which women decide to use the screening services. Therefore; the aim of this current study was to explore the psycho-graphic factors associated to intention to use CCS services among women visiting maternal and child health services. The hypothesis of the study was; women’s intention to use CCS services is a function of Attitude, Subjective norms and PBC.

### Operational definitions

#### Health extension workers (HEW)

Are trained community health workers as a part of achieving primary health care goal. They are tasked to transfer health knowledge and skills to families they serve.

#### Health development army (H.D.A)

These are group of women in the community selected from model families (model households) as health agents. Model households are those who have demonstrated behavior change and improved uptake of high-impact health interventions of HEWs.

#### Elicitation study

It is an exploratory qualitative interview conducted with 15–20 participants selected among target population. It’s done to elicit important psychographic beliefs regarding the behavioral consequences of particular behavior (i.e. CCS use in this case), the significant others and control beliefs for this behavior.

## Methods

### Study design and setting

An institution based cross-sectional study was conducted in *yirgalem* town, Ethiopia from March 1-30, 2017. *Yirgalem* is one of towns located in the South Nations Nationalities and Peoples Region (SNNPR); about 260 KM south of Addis Ababa; the capital city of Ethiopia. According to census 2007; Ethiopia; projection for 2016, the total population of the town was 61,260 with 30,145 were male and 31,115 female of all age categories. The town has five ‘*Kebeles’* (the smallest administrative structure). There were one general hospital, one health center and seven private health institutions in the town.

### Study participants

Women age 30-49 years who were attending maternal and child health services like antenatal care (ANC), postnatal care (PNC) and family planning (FP) services in these health institutions were included. Women who had confirmed cancer of the cervix were excluded from the sample.

### Sample size and sampling techniques

A single population formula for mean difference calculation of finite population was used to calculate sample size as given in the equation; n = (Z_α/2_ ∗*σ*/*d*)^2^. Where; the estimate of predicted variance in intention to CCS was taken to be 50%, the margin of sampling error tolerated (d = 0.05), the value of *Z* score at 95% confidence level (Z_α/2_ = 1.96). This yields a sample of *n* = 384. After adding 10% for non-response; the final sample was 422 women. The single population formula for mean difference was used since the objective of the study was to predict intention to CCS among pregnant women which was measured on summative scales to be treated as continuous variable in the subsequent analyses. Further, since there was no evidence of the value of σ (variance) on similar issue of the current study in Ethiopia); the σ = 50% or 0.5 which yields adequate sample for better inference was used in this study.

There were nine health institutions (one public general hospital, one public health center and seven private clinics) in the town of which only five of them were providing cervical cancer screening services in 2017. Systematic sampling technique was used to select actual participants. Before deciding on the sampling interval (k^th^ value); one-month prior clients flow rate for maternal and child health services was calculated assuming no/low fluctuation flow rates in the next one month of data collection period. There were a total of 902 client flows of all health facilities in the month preceding. Indeed, we would expect the total flow rate during the next one month data collection period to be nearly 902. By dividing 902 to sample size; 902/422=2. So; every other woman visiting maternal and child health clinics in each health facility were approached and exit interview was conducted at each services unit.

### Data collection

Questionnaire was adapted from previous studies [14, 20–22] and modified based on the result of elicitation study. It was developed in English and translated into local languages, ‘*Sidamic’* and ‘*Amharic’* by language experts. Data were collected using interviewer administered basis as an exit interview. In addition to constructs of TPB, the questionnaire covered sociodemographic information, past screening practices, knowledge about CCS and medical and reproductive histories. Refreshment training was given for field workers. Before developing quantitative instrument; we conducted an elicitation study using in-depth interview to explore relevant salient beliefs among a study population regarding perceived outcome expectation of using CCS services, perceived social normative influences and beliefs about personal autonomy in seeking the services. Fourteen women participants were recruited from health institution not included in the actual study. Participants are encouraged to reveal their beliefs regarding benefits using CCS services, the group of people they think would encourage or discourage them to using of CCS services and factors that would either hinder or facilitate their use of CCS services. Furthermore, the cultural relevance and language clarity of the items in the structured questionnaires was explored via pretest which was conducted prior to data collection.

### Measurements and scoring

More specifically, the measurement and scoring technique used in study were adapted from previous study conducted applying the TPB [22]. To this end, intention to use CCS was measured by using four items. Responses ranged from ‘not likely at all’ (1) to ‘very likely’ (5). Composite score was done by summing up all the items.

Direct attitude towards the use of CCS was measured using four items on semantic differential scales (SDS) measuring about the benefit/outcome of using CCS services in the next 3 months on bipolar adjectives (words with opposite meaning). Six items were used to measure behavioral belief with responses ranging from ‘strongly disagree (1) to ‘strongly agree’ (5). Evaluation of CCS belief was measured by asking respondents to evaluate six salient consequences accruing from using CCS services. Each behavioral belief was multiplied by the score for the outcome evaluation to create a new variable (indirect attitude) that represents the weighted score for each behavioral belief.

Four Likert scale items were used to measure direct subjective CCS norm. To assess indirect SN towards CCS, participants were asked seven Likert scale items to indicate the extent to which they thought their health extension workers (HEW), health development army (H.D.A), neighbors, public forum leaders and health workers were likely to appreciate their use of CCS services. Similarly, we weighted each normative belief by the score for motivation to comply belief. Then; the composite scores of indirect SN were created by summing up of the weighted beliefs.

Direct measure of PBC was measured by using four items on bipolar differential scales. High composite score shows strong perceived ability or less difficulty to have CCS services within the specified period of time. Six control belief items were used to measure indirect PBC ranging from unlikely to likely scale and perceived power of control was measured using six items on bipolar Likert scale ranging from ‘strongly disagree’ to ‘strongly agree’ scored on − 2 to + 2 scale response format. The control belief items were multiplied by those of perceived power of control of the beliefs. Summing up of these product scores yields the composite score of indirect perceived behavioral control. In all cases, the higher scores indicate a greater value for all measured TPB constructs to towards the use of cervical cancer screening services (Table 1).

**Table 1:** Summary of measurements and scoring of direct and belief based measures of constructs of TPB of the current study

		Items	Scale	Scoring	Outcome
Dimension (Direct measures)					
1.	Intention (Ii)	4	Likert scale	$\sum \limits_1^4 Ii$	Intention score
2.	Attitude (A)	4	SDS	$\sum \limits_{i=1}^4 Ai$	“A” score
3.	Subjective norms (SN)	4	Likert scale	$\sum \limits_{i=1}^4 SNi$	“SN” score
4.	Perceived behavioral control (PBC)	4	SDS	$\sum \limits_{i=1}^4 PBCi$	“PBC” score
Beliefs based (Indirect measures)					
1.	Behavioral beliefs (bbi)	6	Likert scale	$\sum \limits_{i=1}^6 bbi\ast ebbi$	“IA” score
2.	Evaluation of the beliefs (ebbi)	6	Likert scale		
3.	Normative beliefs (nbi)	7	Likert scale	$\sum \limits_{i=1}^7 nbi\ast mci$	“ISN” score
4.	Motivation to comply (mci)	7	Likert scale		
5.	Control beliefs (cbi) Power of control (pci)	6	Likert scale	$\sum \limits_{i=1}^6 cbi\ast pci$	“IPBC” score

Key: *SDS* Semantic differential scale, *IA* Indirect attitude, *ISN* Indirect SN and *IPBC* indirect PBC

Knowledge about cervical cancer and screening were assessed using 19 items with ‘Yes’ or ‘No’ response format about sign and symptoms, risk factors, methods of prevention, stage of curacy, frequency of screening and eligibility for screening. All items were scored as continuous variables and pulled together where the mean score was computed for further analysis [23]. Practice of CCS (Past behavior experience, PBE): One item was used to ask respondents whether they have ever been screened for CCS by using yes/No approach.

### Data analysis

Data was checked manually for its completeness every day during data collection period. The responses in the completed questionnaire were coded and entered in to Epi-data version 3.1 and exported to Statistical Packages for Social Sciences’ (SPSS) window 21.0 for analysis (v 21.0; IBM Corporation, Armonk, NY, USA). Descriptive Statistical measures like mean and standard deviation were done. The Pearson’s correlation analysis was carried out to examine the association between intention and constructs of theory of planned behavior as bivariate analysis. Similarly; an independent sample t-test and one way ANOVA was carried out to explore the associations between intention and categorical sociodemographic variables. Those variables which have significant associations with intention to CCS at *p* < 0.05 in bi-variate analysis were qualified for multiple regression analysis. Multiple linear regression models were conducted to identify independent factors associated to intention to use cervical cancer screening service. The “enter” regression technique was used to run the analysis. *P*-value < 0.05 was considered to indicate significant association.

## Result

### Socio-demographic characteristics

From 422 participants; 402 respondents completed the interview and producing 95% response rate. The age of the respondents was ranged between 30 to 49 years with mean age of 36.40 ± 4.791 years. The majority 278 (69.2%) were protestant followed by orthodox accounting for 66 (16.4%). Most of the 288 (71.6%) of the respondents were *Sidama* ethnic groups. Majority of the respondents 362 (90%) were married. One hundred fifty-four; 46% of respondents were housewife. About; 191 (47.5%) had completed primary school and 60 (14.9%) were uneducated; who are unable to read and write (Table 2).

**Table 2:** Socio-demographic characteristics of respondents in Yirgalem town, Ethiopia 2017 (*n* = 402)

Variables	Categories	Frequencies	Percent
Age in Years	30–34	150	37.3
	35–39	131	32.6
	40–44	94	23.4
	45–49	27	6.7
Religion	Protestant	278	69.2
	Orthodox	66	16.4
	Muslim	33	8.2
	Catholic	25	6.2
Ethnicity	Sidama	288	71.6
	Amahara	42	10.4
	Oromo	29	7.2
	Gurage	17	4.2
	Wolayita	14	3.5
	Silte	12	3
Marital status	Married	362	90.0
	Widowed	21	5.2
	Single	13	3.2
	Divorced	6	1.5
Occupation	Housewife	185	46
	Self	129	32.1
	Governmental	88	21.9
Monthly income	< 500	77	19.2
	500–999	94	23.4
	> 1000	231	57.5
Educational status	uneducated	60	14.9
	Primary	191	47.5
	Secondary and above	151	37.6

### Source of information, knowledge of cancer and past cervical cancer screening practice

Out of 391 (97.3%) of respondents who have heard about cervical cancer and cervical cancer screening, 166 (42.5%) heard from mass media (radio/TV) and 114(29.8%) from health workers. Regarding knowledge about cervical cancer and screening, 200 (44.2%), 154 (39.4%), 136 (34.95%) and 193(49.4%) knew about symptoms, risk factors for cervical cancer, prevention methods, stages of treatment respectively. With respect to frequency and age of screening, 42(11%) of the respondents knew cervical cancer screening is necessary every 5 years and 90 (23.6%) knew age group mostly affected. For each knowledge items, scores were summed and mean score was computed. Accordingly, only 162 (41.4%) of the respondents were answered above the mean and considered as knowledgeable. Cervical cancer screening experience was low in the area, as only 36 (9.2%) had screened for it.

The mean score of direct attitude, subjective norm and PBC were 14.4 (SD=2.1), 13.71 (SD=1.97) and 13.74 (SD=1.97) respectively and intention with mean score of 13.14(SD=1.7). There was higher attitude score 14.40 (SD=2.1) towards cervical cancer screening among women (Table 3).

**Table 3:** Descriptive statistics for components of theory of planned behavior and intention for women visiting health institution in Yirgalem town (*N* = 402)

S.N	Constructs	Number of items	Scale range	Scale mean	SD	α-value
1	Intention	4	4–20	13.14	1.70	0.69
2	Attitude	4	4–20	14.40	2.10	0.75
3	Subjective norm	4	4–20	13.71	1.97	0.73
4	PBC	4	4–20	13.74	1.97	0.78
5	Total	16	14–80	–	–	

Abbreviation: *PBC* direct perceived behavioral control, *SD* Standard deviation, *α* Cronbach’s alpha

### Relationship between intention, socio demographic factors and measures of TPB constructs

To explore association between dependent and independent variables; all the necessary bi-variate analysis were done. These include; the Pearson’s correlation, an independent sample t-test and one way ANOVA. Unfortunately, all the socio-demographic and pregnancy related variables were not significantly associated with intention to CCS. These variables were occupational status, income level, educational status, number of pregnancy, number of children, and marital status, other risky behaviors like smoking, sexual practice, history of sexual transmitted diseases etc. But the only variable that emerges as significant factors was knowledge about cervical cancer and its screening services.

However, the Pearson’s correlation coefficients showed that all the direct measures of TPB were significantly and positively correlated with each other and with their respective indirect measures. From Table 4 below; the highest and lowest positive correlation was observed between intention and PBC (r = 0.485, *p* < 0.001), and between intention and attitude respectively. Importantly; all indirect measures of TPB were positively and significantly correlated with their respective direct measures. This correlation is required to decide whether to run or not the regression analysis to predict intention using the direct measures. Finally, all indirect measures were positively and significantly correlated with each other. The highest inter-indirect measures correlation was observed between indirect attitude and indirect subjective norm (r = 0.546, *p* < 0.001) (Table 4).

**Table 4:** Pearson’s Correlation between intention, direct and indirect measure of TPB (*n* = 402)

Component	DA	DSN	DPBC	IDA	IDSN	IDPBC Intention
DATT	1					
DSN	0.307**	1				
DPBC	0.328**	0.526**	1			
IATT	0.361**	0.335**	0.357**	1		
ISN	0.152**	0.361**	0.313**	0.698**	1	
IPBC	0.231**	0.408**	0.401**	0.546**	0.487**	1
Intention	0.302**	0.461**	0.485**	0.491**	0.462**	0.430** 1

Abbreviations: *DA* direct attitude, *DSN* direct subjective norm, *DPBC* direct perceived behavioral control, *IDA* Indirect attitude, *IDSN* Indirect subjective norm, *IDPBC* indirect perceived behavioral control. “**” the correlation is significant at *P* < 0.01

### Independent factors associated to intention to cervical cancer screening and interpretations

Prediction of behavioral intention to CCS use was conducted with all direct constructs of TPB and variables that were significant in bi-variate analysis. These are; attitude, subjective norms, perceived behavioral control, knowledge and past behavioral experience (PBE) of cervical cancer screening were entered to multivariate linear analysis. The standardized regression coefficients, PBC was found to be the best factor (β = 0.297, *p* < 0.01) followed by subjective norm (β = 0.248, p < 0.01).This indicates; a unit positive change in women’s perception of ability to control over circumstances that inhibit them from using CCS will increase intention to use CCS by 29.7% keeping other conditions constant. Women who perceive significant others will approve of their using CCS services will have 24.8% higher intention to use CCS than their counterparts. Similarly, a unit-positive change in women’s attitude toward the advantage associated with the use of CCS services will increase the individual’s intention to use it by 11% provided that all the other factors kept unvaried. In this study, attitude was found to be the least factor associated to intention to CCS use (β = 0.11, *p* < 0.014).

In this study; the past behavioral experience (PBE) of using CCS services positively predicted intention to use CCS (β = 0.098, *P <* 0.02). Intention to use CCS services will significantly be increased by 9.8% for those women who have an experience of using the services (Table 5).

**Table 5:** Independent factors associated to behavioral intention to cervical cancer screening, *Yirgalem* town, southern Ethiopia, 2017 (*n* = 402)

Variables	Standardized β	*P*-value	95% CI for β
PBC	0.297	0.01	[0.172, 0.340]
Subjective norm	0.248	0.01	[0.131, 0.301]
Attitude	0.110	0.01	[0.018, 0.160]
PBE	0.098	0.02	[0.093, 1.001]
Knowledge score	0.145	0.01	[0.047, 0.170]

Abbreviations: *PBC* perceived behavioral control, *PBE* past behavior experience

## Discussion

The aim of this study was to determine intention to use cervical cancer screening and its associated psychometric and socio-demographic factors among women. To this regard, the results of this study revealed none of socio-demographic variables significantly predicted intention to the contrary to previous studies conducted in Iran and somewhere else [18, 24]. This may be due to the setting in which the study was conducted.In this study, 97.3% of respondents have awareness about the existence of cervical cancer screening services in Ethiopia. This finding is high compared to community-based studies done in Ethiopia in 2016 and 2010 [10, 25]. This difference may be due to the population under study as those who visit health institution may have higher chance of getting access to information than their counterparts. The recent strong efforts for cancer prevention and control program being done by Ethiopian government might have a contribution to this difference. The National Action Plan on the Prevention and Control of Chronic Non-Communicable Diseases has addressed strategies to scale up availability and accessibility of health services for cervical cancer care through effective participatory prevention and control methods with faith organization, traditional healers, community, non-governmental organization and private institutions since 2015 [26].

It’s known that knowledge on causal factors and methods of prevention is essential in the prevention of cervical cancer. Knowledge influences individuals decision making in that knowing these factors can influence someone avoids or overcome them and hence escapes from acquiring the disease. In our study; the overall knowledge of the disease signs, symptoms, risk factors and respective prevention methods was as low; 162 (41.4%). Hence, public health education on these issues for women has paramount importance. However, it is relatively higher than the finding of previous study done in Ethiopia revealing only 24.8 and 27% of the respondents know about sign and symptom, and main risk factors of cervical cancer respectively [10]. The discrepancy might be due to the difference in time of study as our study was done latter after due attention has been given to cervical cancer screening services through ministry of health initiatives [7].

In our study; being knowledgeable about cervical cancer and screening was significantly and positively associated with cervical cancer screening intention. Knowledge is important and necessary especially for rational decision of any health behavior even though; it is not sufficient. This finding suggests; knowledge might have positive impact on attitude to influence an individual intend for screening. Similar finding was reported from related studies done in China [27] and Mandalay [28].

In this study, only 9.2% of the women reported they had screening for cervical cancer. However, it’s higher than the country wide prevalence of CCS; which was less than 1% by 2008 [7]. The difference might be attributed to time variation and strong initiatives being undertaken by the government in the recent time. Ethiopia has adopted a comprehensive National Action Plan on the Prevention and Control of Chronic Non-Communicable Diseases (NCDs), including cancer in 2015. The country planned a nation-wide scale up of the screening and treatment for cervical cancer into over 800 health facilities (one health facility per district). The National Cancer Committee (NCC) was organized at federal level which has been chaired by the late First Lady of Ethiopia [26]. Furthermore; the social desirability bias (SDB) inherent to self-reports of being screened for cervical cancer might also have contributed to this difference.

Similar to studies conducted in Latinas and Iran [18, 19], the current study has identified that the CCS experiences was associated with cancer screening intention. To this regard, Ajzen; the author of TPB; has explored that the role external variables (e.g. past behavior experiences) in influencing intention through proximal TPB constructs [14]. Further; study conducted in Ethiopia and Tanzania (on condom and VCT use behavioral intention) showed conflicting results on the effects of past experiences on behavioral intention [22, 29, 30].According to Ajzen, the intention to perform a behavior is a function of attitude, subjective norms, and PBC and that this varies according to the behavior, the population under study, the context and time in which the study conducted [14]. This study revealed that intention to use cervical cancer screening among women was mostly influenced by perceived behavioral control followed by subjective norm to cervical cancer screening. Attitude was identified as least significant predictor constructs of theory of planned behavior. This result is consistent with a study conducted among Latinos in which perceived behavioral control was strongest predictor of intentions; followed by subjective norms to be screened for cervical cancer [19, 20]. The result of our study implies that directing interventions strategies that can increase women’s sense of control over circumstances around cervical cancer screening could have potential impact on screening intention and behavior.

Subjective norm was the second strongest associated factor of intention. This implies; those women who were more strongly endorsed that their important others expected them to have cervical cancer screening expressed stronger intention. As such, considering interventions addressing normative dimension could have an effect on screening behavior. Similar finding was reported form study conducted on intention to use CCS services [18]. However; study done somewhere else revealed that subjective norm did not have significant effect on intention to seek cervical cancer screening [24]. This gap could be attributed to the difference in the context where the study undertaken.

In this study, attitude was the least significantly and positively associated attribute with cervical cancer screening intention. In line with this result; a study done in London revealed, that attitude is the least significance and positively associated with intention to CCS [31]. However, other study conducted in Uganda found that, attitude is the best predictor followed by subjective norm with intention towards cervical cancer screening [21]. This could be attributed to the difference in the context/circumstances under which the behavior is to take place and the region/place where the study was conducted as already mentioned above [14].Although, our study has addressed pressing public health issues by applying effective health behavioral theory; it has inevitably few limitations. The study did not employ experimental design to establish the causal effect relations among these psychographic variables. In addition, the social desirability bias might affect the accuracy of the data through its influence on individuals’ attitude and intention.

## Conclusions

Overall; the study revealed that the behavioral intention to use CCS services was a function of the psychographic variables; attitude, perceived social pressure and PBC confirming the hypothesis of our study. The PBC was found to be most important factor associated to behavioral intention. None of the socio-demographic variables were significantly associated to intention. Health behavior change interventions should focus on increasing knowledge, perceived power that enable women to evaluate their control belief positively and empower them to develop ability against social norms that could compete with the use of CCS services and build an attitude that support the behavior. Further study employing longitudinal design should be conducted to see the translation of behavioral intention to the actual behavior thereby establishing causal effect relation.

## Abbreviations

ANC: Antenatal care
CCS: Cervical cancer screening
FP: Family planning
H.D.A: Health development army
HEW: Health extension workers
PBC: Perceived behavioral control
PBE: Past behavior experience
PNC: Postnatal care
SNNRP: South Nation Nationality and Peoples Region
SPSS: Statistical package for social sciences
TPB: Theory of planned behavior
TRA: Theory of Reasoned Action
USA: United States of America
